# Foundation models in ophthalmology: opportunities and challenges

**DOI:** 10.1097/ICU.0000000000001091

**Published:** 2024-11-04

**Authors:** Mertcan Sevgi, Eden Ruffell, Fares Antaki, Mark A. Chia, Pearse A. Keane

**Affiliations:** a Institute of Ophthalmology, University College London; b Moorfields Eye Hospital NHS Foundation Trust; c Institute of Health Informatics; d Centre for Medical Image Computing, University College London; e NIHR Biomedical Research Centre at Moorfields Eye Hospital NHS Foundation Trust, London, UK; f The CHUM School of Artificial Intelligence in Healthcare, Montreal, Quebec, Canada

**Keywords:** foundation models, large language models, large vision models, medical artificial intelligence, multimodal models

## Abstract

**Purpose of review:**

Last year marked the development of the first foundation model in ophthalmology, RETFound, setting the stage for generalizable medical artificial intelligence (GMAI) that can adapt to novel tasks. Additionally, rapid advancements in large language model (LLM) technology, including models such as GPT-4 and Gemini, have been tailored for medical specialization and evaluated on clinical scenarios with promising results. This review explores the opportunities and challenges for further advancements in these technologies.

**Recent findings:**

RETFound outperforms traditional deep learning models in specific tasks, even when only fine-tuned on small datasets. Additionally, LMMs like Med-Gemini and Medprompt GPT-4 perform better than out-of-the-box models for ophthalmology tasks. However, there is still a significant deficiency in ophthalmology-specific multimodal models. This gap is primarily due to the substantial computational resources required to train these models and the limitations of high-quality ophthalmology datasets.

**Summary:**

Overall, foundation models in ophthalmology present promising opportunities but face challenges, particularly the need for high-quality, standardized datasets for training and specialization. Although development has primarily focused on large language and vision models, the greatest opportunities lie in advancing large multimodal models, which can more closely mimic the capabilities of clinicians.

## INTRODUCTION

The advancement of artificial intelligence in ophthalmology has been significantly propelled by deep learning techniques, which analyse eye images to detect patterns and anomalies, thereby significantly enhancing diagnostic and prognostic capabilities [1]. Despite their effectiveness, a main constraint for such artificial intelligence models is that they typically require vast amounts of labelled data for model training, often not obtainable. 

**Box 1:**
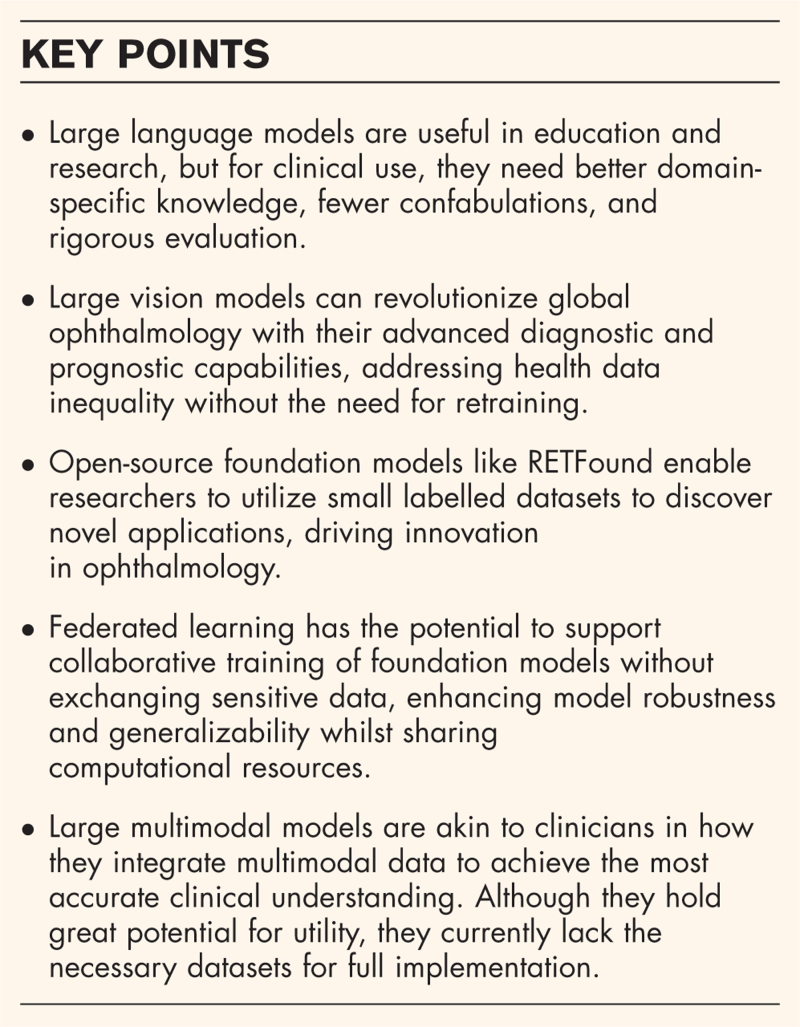
no caption available

**FIGURE 1:**
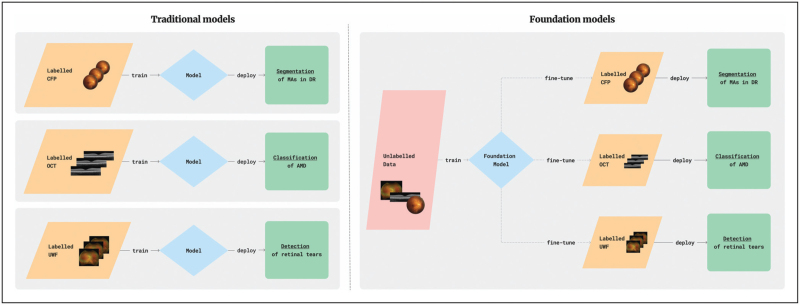
Schematic representation of training traditional deep learning models and foundation models. The differences between training traditional deep learning (DL) models and foundation models (FM) are highlighted. Traditional DL models typically require labelled datasets and are trained for specific tasks. In contrast, foundation models are usually trained once on unlabelled data and subsequently fine-tuned for a variety of tasks and modalities, such as segmentation, classification, and object detection. CFP, colour fundus photo; DN, diabetic retinopathy; MA, microaneurysm; OCT, optical coherence tomography; UWF, ultra-wide field. Adapted from [4], licensed under a Creative Commons Attribution 4.0 International (CC BY 4.0) Licence.

More recently, the widespread use of foundation models in healthcare has marked a significant step towards general medical artificial intelligence (GMAI). Coined by researchers at Stanford, ‘foundation model’ refers to deep learning models that provide a versatile base of knowledge adaptable for various specific purposes, unlike traditional artificial intelligence systems designed for specific tasks (Fig. 1) [2,3]. Enabled by innovative approaches such as self-supervised learning (SSL) and vision transformers (ViTs), foundation models are trained on large, diverse, unlabelled datasets and fine-tuned with smaller labelled datasets for specific medical use cases. This approach allows foundation models to achieve the same accuracy as traditional models but with fewer labelled examples [4].

In this review, we examine the current status and opportunities of foundation models in ophthalmology, including large language models (LLMs), large vision models (LVMs) and large multimodal models (LMMs). Notably, we discuss RETFound, the first LVM for ophthalmology and its current use cases and future opportunities. We also explore the key next challenges to advance foundation models in ophthalmology.

## LARGE LANGUAGE MODELS

Among the most well known types of foundation models are LLMs, known for their capacity to understand and generate human-like text [5,6]. Training these LLMs involves processing extensive text data from diverse sources, often encompassing billions of words, which enables them to grasp nuanced and complex language patterns [5].

**FIGURE 2:**
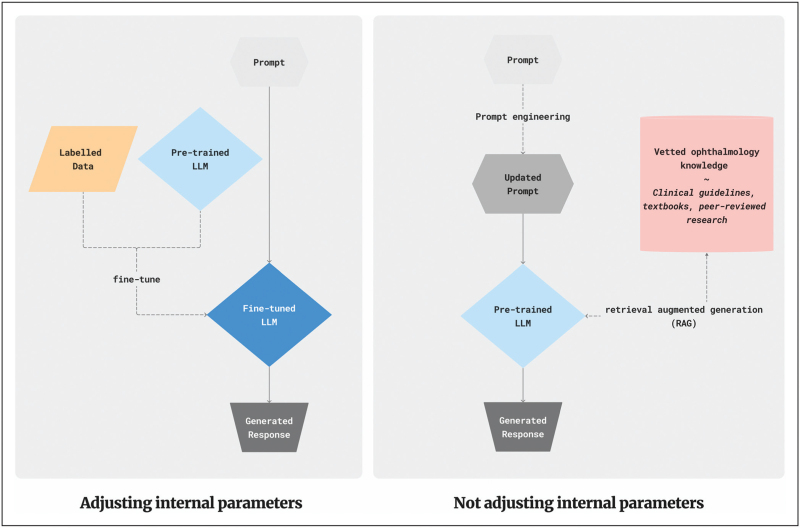
Methods of specializing large language models. The various techniques used to tailor LLMs for specific applications, including fine-tuning, prompt engineering, and retrieval-augmented generation (RAG) are illustrated. Fine-tuning involves adjusting the internal model parameters to improve performance on a specific task, while prompt engineering and RAG do not alter the model parameters but instead enhance the model's output through different approaches.

To enhance these models, newer iterations require larger and more up-to-date text corpora for training, in addition to vast amounts of computational power [7]. Within ophthalmology, we often lack the resources and data to train a domain-specific LLM from scratch. Instead, it is more beneficial to specialize existing LLMs using methods such as fine-tuning, prompt engineering, and retrieval-augmented generation (RAG) as seen in Fig. 2[8–11].

Fine-tuning involves updating the internal parameters of the LLM using small labelled datasets, to equip the model with specific domain knowledge. Within the context of ophthalmology, an LLM can be fine-tuned using validated question and answer (QnA) pairs. This process can involve providing accurate responses to clinical management questions, such as the treatment protocols, for example, acute angle-closure glaucoma [12]. When updating the internal parameters, fine-tuning can be approached in two main ways, with a frozen or unfrozen backbone. Fine-tuning with a frozen backbone means that the core parameters of the model remain unchanged or ‘frozen’, and only the top parameters, known as the head, are updated [13]. This approach is computationally efficient and reduces the risk of overfitting, which is when an artificial intelligence model replicates the training data without learning the underlying patterns. However, with a frozen backbone, the ability to adapt to highly specialized tasks is limited because the core parameters cannot be modified. Conversely, fine-tuning with an unfrozen backbone involves updating the entire model, including the core parameters. This allows for greater adaptability and often results in higher accuracy on domain-specific tasks, as the model can fine-tune all its parameters end-to-end. The trade-off is that this approach requires more computational resources, takes longer to train, and has a higher risk of overfitting because of the extensive parameter updates [13]. Both of these approaches typically require technical coding skills, however, recent advancements on platforms such as Google's Vertex AI allow for fine-tuning LLMs without the need for coding knowledge [10].

Other methods of specialization focus on optimizing the outputs of the LLM rather than altering its parameters. These approaches enable the model to act as an agent, capable of retrieving domain-specific information [14]. Given the propensity for LLMs to confabulate information or provide outdated knowledge with opaque reasoning processes, RAG has emerged as a promising solution that allows LLMs to pull from external databases when generating a response [11]. Such external databases could include peer-reviewed articles, clinical trials, guidelines, and electronic health records (EHR). This approach enhances the accuracy and credibility of LLM outputs, especially for knowledge-intensive tasks seen in healthcare, by allowing continuous updates and integration of domain-specific information. For example, practical use cases of LLMs with RAG include creating virtual teachers that generate Q&A from clinical guidelines, and summarize research articles [15^▪^]. Platforms like OpenAI allow users to create their own custom GPT models. These models can use RAG to incorporate additional documents and perform web searches for more accurate and comprehensive responses [16].

Similar to RAG, another approach to LLM specialization is prompt engineering. This technique steers the model's responses by designing and structuring input prompts to guide the model towards producing more accurate and relevant outputs [17]. By providing specific contexts or framing questions in particular ways, prompt engineering helps the model leverage its existing knowledge more effectively, ensuring that generated responses align closely with the desired domain-specific information [14,18]. For prompt engineering, there are several different prompting strategies that can be applied, depending on what is required [17,19]. For example, the simplest approach is zero-shot or direct prompting is when the prompt used to interact with the LLM does not contain examples or demonstrations, for example, ‘Give me a list of causes of red eye’, Another type of prompt engineering is role prompting, which involves assigning the LLM a specific role, for instance ‘You are a clinical triage assistant, please explain to the patient the causes of red eye’. In this way, the prompt provides the LLM with situational context and a better understanding of what style of language it should use for its audience [17]. Through methods like one-shot or few-shot prompting, an LLM is provided with the ideal output response structure, to help guide its responses if they are required to be in a specific format such as a clinical letter or report. Finally, chain-of-thought (CoT) prompting encourages the LLM to explain its reasoning step-by-step, which is particularly important in healthcare to understand the reasoning behind the model's clinical responses [17]. For instance, ‘Explain your thought process for diagnosing the cause of red eye in a patient presenting with these symptoms’.

Before specializing a foundation model, such as an LLM, it is common to first use a linear probe to evaluate the quality of the patterns and features learned by the foundation model. A linear probe involves training a simple classifier, such as logistic regression, on top of the pretrained foundation model to perform a specific task. This method helps determine how well the pretrained features can support the task with minimal additional learning. If a linear probe performs well, it suggests that the model's pretrained knowledge is robust and effective for the given task.

These specialized LLMs often outperform traditional machine learning models, even when using very small labelled datasets, making for a more effective use of the limited data available in hospitals [4]. Many popular LLMs such as Gemini and GPT-4, have undergone general medical specialization using these techniques, exemplified by GPT-4 Medprompt and Med-Gemini. These achieve medical specialization through a combination of self-training via web search, fine-tuning with customised encoders and prompt engineering using CoT prompting [18,20^▪▪^].

Initially, most evaluations of LLMs in ophthalmology have focused on assessing the performance of out-of-the-box models through a variety of specific tasks, without medical specialization [21]. These tasks include answering patient queries, drafting operative notes, triaging clinical cases, analysing imaging reports, and responding to clinical examination questions [22–27,28^▪^]. More recent research has begun to outline and explore the benefits of specializing LLMs specifically to ophthalmology encompassing use-cases in medical education, workflow improvement and clinical assistance [12,29,30]. LLMs for medical education could summarize clinical guidelines and academic articles, as well as acting as a virtual patient to which questions can be asked to aid learning [15^▪^,^16^]. Meanwhile, report generation and streamlining EHR documentation are ways in which LLMs could improve workflow management [29,31].

For clinicians, LLMs could also aid in summarizing clinical content, assist with making referrals and provide suggestions based on the medical history. As reliance on tertiary-level eye care services increases, these types of LLMs are likely to be increasingly useful [32]. For example, they may be integrated into face-to-face clinical encounters or into virtual clinic services, complementing tele-ophthalmology efforts with the potential to transform patient care in underserved areas [33]. In low-income and middle-income countries (LMICs), there is a significant shortage of clinicians, which exacerbates the challenges of providing adequate eye care [34]. Therefore, implementing LLMs in these regions could yield substantial benefits by augmenting the limited human resources, improving access to eye care, and enhancing the overall quality of patient care [29,35]. Depending on their use cases, such as a patient-facing application for diagnosing diseases, LLMs could be considered as medical devices. In these cases, the deployment of such LLMs would require regulatory approval, necessitating rigorous testing and validation to ensure their safety and efficacy in clinical settings [36].

## LARGE VISION MODELS

In addition to language processing, foundation models can be trained to have vision capabilities, which can be useful for analysing and interpreting medical images. These models, known as LVMs, are especially valuable in ophthalmology, a field heavily reliant on imaging. However, while imaging data is routinely collected in ophthalmology, it is often unlabelled as it is not typically accompanied by formal reports, as seen in radiology. By employing techniques such as SSL, we can harness this extensive amount of unlabelled image data to train LVMs effectively [37^▪▪^].

Developed by researchers at Moorfields Eye Hospital and UCL, RETFound serves as the first LVM for ophthalmology. It was trained separately on colour fundus photography (CFP) and optical coherence tomography (OCT), resulting in two versions of the model. When fine-tuned for specific downstream tasks, using small labelled datasets, RETFound was employed for diagnosis and prognosis of ocular diseases and the prediction of systemic diseases, where it outperformed other supervised and self-supervised models. As an open-source model, RETFound is accessible for other groups to explore and fine-tune for new tasks, such as image segmentation [38].

One potential research avenue in ophthalmology is the development of LVMs trained on 3D images, a technique being explored in other medical specialties [39^▪▪^]. At present, LVMs like RETFound tend to only utilize a select number of 2D B-scans from an OCT volume during training, such as the central B-scan [40^▪▪^]. Therefore, creating a model that utilizes the entire OCT volume has the potential to provide greater spatial information and thus more accurate diagnosis. However, within ophthalmology, there is a notable lack of pretrained 3D models to test this hypothesis [41].

Similarly, research is being conducted to better understand the benefits of training LVMs on multiple imaging modalities, with VisionFM and EyeFound as being notable examples [30,42]. Both models were trained on large datasets, including modalities such as OCT, CFP, fundus fluorescein angiography, in addition to B-ultrasound, external eye photos and slit lamp photos. Incorporating these modalities is valuable as it covers a broad spectrum of ophthalmic conditions across multiple sub-specialties. However, whilst these models report high accuracy in diagnosing ocular diseases showing promising results, it is important to note that these models are currently detailed in preprint, and further development and rigorous validation are necessary to fully confirm their capabilities.

## LARGE MULTIMODAL MODELS

Despite the promise of LLMs and LVMs, to truly assist clinicians, it is essential to develop models that can integrate various types of data, such as text and images. Developing such LMMs marks a step towards GMAI, mirroring how clinicians combine multiple data modalities for a comprehensive understanding. Consequently, more recent foundation model research has emphasized developing LMMs with multimodal capabilities.

**FIGURE 3:**
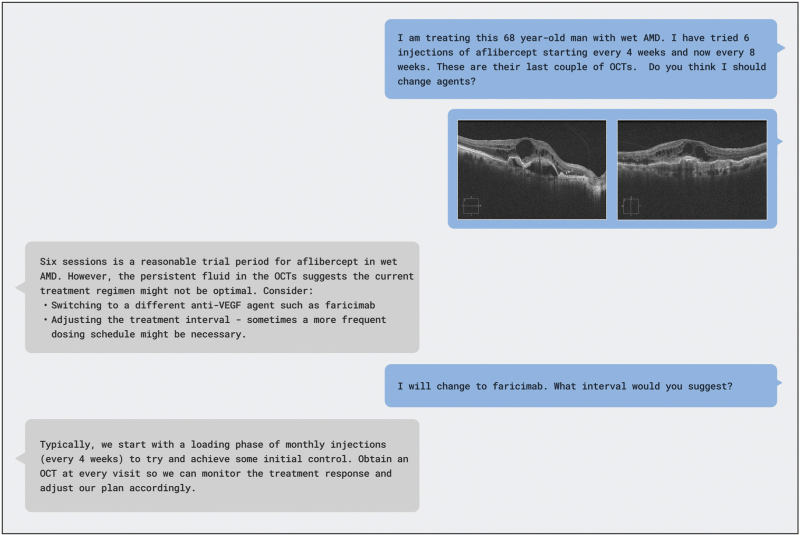
Visual question and answer example scenario involving an ophthalmologist using a large multimodal language model for treating wet age-related macular degeneration. The LMM interprets OCT (optical coherence tomography) images of a patient with wet age-related macular degeneration, offering guidance on treatment adjustment. The model also responds to follow-up questions. Images are from [43] licensed under a Creative Commons Attribution 4.0 International (CC BY 4.0) Licence. LMM, large multimodal models.

One such model is Google's Med-Gemini, an LMM adapted from Gemini on diverse medical text and imaging data from various medical specialties, including CFP images from a EyePACS diabetic retinopathy dataset. Using these capabilities, Med-Gemini was developed to support a range of different clinical tasks including disease classification, image report generation, and polygenic risk prediction [20^▪▪^,^39^^▪▪^]. Med-Gemini also performs visual question answering (VQA), where the model responds to visual prompts such as fundus photos and engages in dialogue with the user regarding the management of conditions. For instance, in ophthalmology, the LMM can diagnose a condition based on the medical history provided through text and clinical images as demonstrated in Fig. 3[43].

During evaluation on ophthalmology tasks, Med-Gemini was shown to outperform the out-of-the-box Gemini Ultra in several experiments, but for the task of diabetic retinopathy lesion detection, it did not surpass the accuracy of the supervised learning model. Similar projects have looked to evaluate out-of-the-box LMMs like Google's Gemini Pro, on tasks such as on interpreting OCT B-scans, however, these models have similarly failed to reach high accuracy compared with supervised models [44].

Another LMM with ophthalmology capabilities is OphGLM, fine-tuned from ChatGLM using fundus image–text pairs generated from medical–patient conversations [45]. This model was utilized for a variety of downstream tasks, including generating image descriptions, explaining causes and symptoms, and guiding diagnosis and examination procedures for multiple ocular diseases. Despite showing promise, no formal metrics for the model's full pipeline performance were provided; only examples of output dialogues were shared. Therefore, further clinical evaluation is required to determine its potential value within ophthalmology.

Moreover, it is notable that neither Med-Gemini nor OphGLM were initially trained on ophthalmology data, and only later fine-tuned. Consequently, at their core, these foundation models are not specifically designed as ophthalmology-focused LMMs. We, therefore, speculate that better results could be achieved if LMMs were trained on, at least partially, ophthalmology-specific image–text pairs, during both initial training and fine-tuning. This approach, however, would require large, high-quality, ophthalmology-specific datasets, which are currently lacking in the field, as noted by the authors from the OphGLM paper [45].

## CHALLENGES

Despite these promising steps, the development of foundation models in ophthalmology are confronted with significant constraints. One of the main challenges is the availability of vast ophthalmology-specific datasets needed for foundation model training, as collecting and maintaining high-quality data is costly and time-consuming [21]. This often leads researchers to rely on existing foundation models, such as Gemini, which are trained on general information but can be fine-tuned with ophthalmology-specific data [39^▪▪^]. Models like RETFound, however, incorporate ophthalmic data during both the training and fine-tuning phases with promising results, which demonstrates the substantial advantages of having ophthalmology-specific data integrated throughout the training process [40^▪▪^].

Another significant challenge in developing foundation models for ophthalmology is the immense computational resources required. Building foundation models from scratch requires expensive GPUs, and with each new version, the costs rise because of the increased computational demands [46]. This often makes foundation model training feasible only for large institutions and companies that can afford the necessary infrastructure and resources, again leading researchers to rely on existing pretrained general purpose foundation models, like Gemini and GPT-4.

Given these challenges, it is important to promote the development and use of open-source ophthalmology foundation models, such as RETFound, which can be fine-tuned by other researchers and hospitals using their own datasets. This approach not only democratizes access to advanced artificial intelligence tools but also allows researchers to leverage their limited amounts of labelled data to tailor models to local populations, enhancing their effectiveness and relevance in diverse clinical settings [47].

Another promising solution to overcome these challenges is federated learning, which enables collaborators to share data and resources, thereby mitigating high costs and computational demands [48]. By distributing computational tasks among research collaborators, federated learning helps to alleviate major computational constraints. Additionally, federated learning supports the use of datasets from hospitals to train models without directly sharing data, thereby expanding the pool of available training data without encountering information governance issues. Although this technique has been successfully used to train deep learning models in ophthalmology, using data from multiple centres, there is currently a lack of research around the plausibility of training foundation models using federated learning. Therefore, the next challenge could be to leverage federated learning to build ophthalmology foundation models [49^▪^]. We can also consider using platforms such as FedEYE and Bitfount to provide user-friendly federated learning environments, allowing centres without technical expertise to contribute their data for model training [50,51].

A final hurdle to developing foundation models for ophthalmology is that of regulation. However, recommendations for artificial intelligence as a medical device (AIaMD) are less clear compared with medical devices and drugs [52]. AIaMD is already used in ophthalmology, with research underway to explore its applications in ophthalmic imaging [52]. As we move forward, we need to consider different regulatory requirements for foundation models, especially for integrating language models and vision models [53,54]. Copyright infringement is currently a heated topic in the media [55]. Ensuring the provenance of the data used to train clinical LLMs and establishing clear regulations will be crucial [56].

## CONCLUSION

In summary, foundation models in ophthalmology present an array of promising opportunities and notable challenges. The advancements in LLMs are particularly exciting because of their ability to mimic human-like conversation and provide valuable insights despite current constraints in specific domains such as ophthalmology. The ongoing efforts to improve these models by training on broader medical data and employing fine-tuning methods are encouraging, as they aim to reduce confabulations and enhance model reliability.

Similarly, the potential of vision models in ophthalmology is immense. These models offer advanced diagnostic and prognostic capabilities, and their adaptability to various tasks without the need for retraining is poised to revolutionize global ophthalmology. This is especially crucial in addressing health data inequality. The advent of open-source models like RETFound allows researchers worldwide to experiment and discover novel applications, further driving innovation in the field. Moreover, federated learning offers a promising solution for researchers to train foundation model models collaboratively without exchanging sensitive data, helping to build larger, more diverse foundation models. This collaborative approach not only expands the pool of training data but also enhances the robustness and generalizability of the models, while reducing the computational burden on a single institution.

Lastly, LMMs hold significant promise by integrating various modalities in a manner akin to a clinician's approach, moving closer to achieving GMAI. This capability could fundamentally transform healthcare delivery and the interaction between patients, healthcare professionals, and artificial intelligence systems. However, to make this progress, there is a pressing need for high-quality, standardized datasets specifically for model training and medical specialization. As these technologies continue to evolve, they are likely to play a pivotal role in shaping the future of ophthalmology and healthcare at large, offering more precise, accessible, and efficient care for patients globally.

## Acknowledgements

*None.*

### Financial support and sponsorship

*None.*

*Funding: E.R. is supported by the UCL UKRI Centre for Doctoral Training in AI-enabled healthcare systems Studentship (EP/S021612/1) F.A. is supported by the Fonds de recherche du Québec – Santé (FRQS). M.C. is supported by a General Sir John Monash Scholarship. P.K. is supported by a UK Research & Innovation Future Leaders Fellowship (MR/T019050/1).*

### Conflicts of interest

*There are no conflicts of interest.*

## REFERENCES AND RECOMMENDED READING

Papers of particular interest, published within the annual period of review, have been highlighted as:

▪ of special interest
▪▪ of outstanding interest
